# Involvement of plant endogenous ABA in *Bacillus megaterium* PGPR activity in tomato plants

**DOI:** 10.1186/1471-2229-14-36

**Published:** 2014-01-25

**Authors:** Rosa Porcel, Ángel María Zamarreño, José María García-Mina, Ricardo Aroca

**Affiliations:** 1 Departamento de Microbiología del Suelo y Sistemas Simbióticos, Estación Experimental del Zaidín (EEZ-CSIC), Profesor Albareda 1, 18008 Granada, Spain; 2 CIPAV TimacAGRO International-Roullier Group, Polígono Arazuri-Orkoien, c/C no. 32, 31160-Orkoien, Navarra, Spain

**Keywords:** Abscisic acid, *Bacillus megaterium*, Ethylene, Hormones, PGPR, *Solanum lycopersicum*, Rhizobacteria

## Abstract

**Background:**

Plant growth-promoting rhizobacteria (PGPR) are naturally occurring soil bacteria which benefit plants by improving plant productivity and immunity. The mechanisms involved in these processes include the regulation of plant hormone levels such as ethylene and abscisic acid (ABA). The aim of the present study was to determine whether the activity of *Bacillus megaterium* PGPR is affected by the endogenous ABA content of the host plant. The ABA-deficient tomato mutants *flacca* and *sitiens* and their near-isogenic wild-type parental lines were used. Growth, stomatal conductance, shoot hormone concentration, competition assay for colonization of tomato root tips, and root expression of plant genes expected to be modulated by ABA and PGPR were examined.

**Results:**

Contrary to the wild-type plants in which PGPR stimulated growth rates, PGPR caused growth inhibition in ABA-deficient mutant plants. PGPR also triggered an over accumulation of ethylene in ABA-deficient plants which correlated with a higher expression of the pathogenesis-related gene *Sl-PR1b*.

**Conclusions:**

Positive correlation between over-accumulation of ethylene and a higher expression of *Sl-PR1b* in ABA-deficient mutant plants could indicate that maintenance of normal plant endogenous ABA content may be essential for the growth promoting action of *B. megaterium* by keeping low levels of ethylene production.

## Background

There are numerous reports of plant growth and yield stimulation by beneficial soil microorganisms [1-4]. A wide range of microorganisms which live in the soil are able to establish symbiotic and non-symbiotic associations with their host plants [5]. These microorganisms play important functions in the soil which include: (1) controlling the adverse effects of pathogens on plant growth, (2) alleviating negative effects of soil stresses on plant growth and yield production, (3) biofertilization, (4) enhancing root growth, and (5) rhizoremediation [6-9].

Plant growth-promoting rhizobacteria (PGPR) are among the most effective and best studied soil microorganisms which can promote plant performance. PGPR can be classified as extracellular bacteria (existing in the rhizosphere, on the root surface or in the spaces between cells) and intracellular bacteria (mainly N_2_ fixing bacteria) [5]. The action mechanisms of PGPR can be also divided into direct and indirect ones. Biofertilization, stimulation of root growth, rhizoremediation and plant stress control are the direct mechanisms. On the other hand, the mechanisms of biological control by which rhizobacteria can promote plant growth indirectly, by reducing the level of disease, include antibiosis, induction of systemic resistance and competition for nutrients and niches [8]. Hormones such as auxins, ethylene, gibberellins, (+)- abscisic acid (ABA) and cytokinins regulate plant growth and development [10,11]. Plant hormones are chemical messengers that affect the plant’s ability to respond to its environment. They are organic compounds that are effective at very low concentration and are usually synthesized in one part of the plant and transported to another location. Each plant response is often the result of two or more hormones acting together. Because hormones stimulate or inhibit plant growth, they are also referred as plant growth regulators. Plant-growth-promoting bacteria that contain the enzyme 1-aminocyclopropane-1-carboxylate (ACC) deaminase facilitate plant growth and development by decreasing plant ethylene levels. ACC is the precursor for the production of ethylene, whose amounts are increased under stress, affecting adversely plant growth and yield production. Hence, ethylene is one of the hormones regulating plant growth under different conditions including stress [8]. Ethylene is a plant growth regulator essential for normal growth and development in plants. However, apart from this key function, ethylene also acts as a stress hormone when plants are exposed to salinity, drought, waterlogging, heavy metals or pathogens [12].

ABA plays an important role in many physiological processes in plants. This hormone is necessary for regulation of several events during late seed development and is crucial for the response to environmental stresses such as desiccation, salt and cold. Abscisic acid controls plant growth and inhibits root elongation [13], which means that there is a negative correlation between growth and the endogenous ABA content of plants [14]. Despite this, ABA-deficient plants are usually smaller than wild-type (wt) plants [15]. It has been reported that some bacterial species that interact with plants or live in the soil, synthesize ABA and other phytohormones such as indole-3-acetic acid (IAA), gibberellic acid, zeatin (cytokinin) and ethylene [16-18]. Some species from the genus *Bacillus* have been described not only as plant growth promoters but also as a biological control agents of diseases [19,20]. So far, although impacts of one specific PGPR on ABA relations of *Pisum sativum* have been studied [21], no studies have been conducted to explain how endogenous levels of ABA could affect the PGPR function of *Bacillus*. In our study, tomato was chosen as the host plant. Tomato has a number of well-known ABA pathway mutants and represents an appropriate model for studying the role of endogenous ABA in plants responses to PGPR. The ABA-deficient tomato mutants *flacca* and *sitiens* and their near isogenic wild-type parental line were used in this study. Previous research has shown that these mutants have residual ABA levels (no more than 8% of the wild-type plants) [22] and are unable to increase their ABA levels when plants are exposed to stress [23].

A PGPR from degraded soil in southern Spain isolated by our group and identified as *Bacillus megaterium*[24] has been used in this study. Marulanda et al. [25] analyzed how this PGPR strain may influence two crucial components of plant salt tolerance such as root hydraulic characteristics and aquaporin regulation in maize plants. Maize plants inoculated with *Bacillus megaterium* subjected to salt stress, showed higher biomass production, lower necrotic leaf area and higher root hydraulic conductance than non inoculated control plants [25]. In previous studies carried out by the same group it has been showed that this *Bacillus megaterium* strain was able to increase drought resistance in plants growing under water-limited conditions [26] and to increase plant growth under nutrient deficiency conditions [24]. The aim of the present study was to determine whether the activity of *Bacillus megaterium* PGPR is affected by the endogenous abscisic acid (ABA) content of the host plant.

## Methods

### Experimental design

The experiment consisted of a randomized complete block design with two inoculation treatments: (1) non-inoculated control plants and (2) plants inoculated with the PGPR *Bacillus megaterium* strain which was isolated by Marulanda-Aguirre et al. [24], and two plant ABA line treatments: wild type and an ABA-deficient line (*flacca* or *sitiens*). There were ten replicated plants per treatment (one plant per pot). The plants were cultivated under well watered conditions throughout the entire experiment. Two different sets of experiments were carried out: one with wild type (cv Rheinlands Ruhm) and *sitiens* plants (from January to March) and the second with wild type (cv Ailsa Craig) and *flacca* plants (from March to May). All determinations (except biomass production that was measured in samples taken from both experiments) were carried out on samples taken from the second set of experiments.

### Soil and biological materials

Peat and vermiculite mixture (1:1, v/v) was sterilized (120°C for 20 min). Seeds of tomato (*Solanum lycopersicum*) *sitiens* (LA0574) and its parental isogenic cv Rheinlands Ruhm, as well as *flacca* (LA3613) and corresponding parental isogenic cv Ailsa Craig, were obtained from the Tomato Genetics Resource Center (TGRC) at the University of California, Davis, CA, USA. The seeds were sterilized in a 70% ethanol solution for 2 min, then 50% sodium hypochlorite solution for 8 min and finally washed several times with sterile water to remove any trace of chemicals that could interfere with seed germination. The seeds were placed on sterile vermiculite at 25°C to germinate and 10-d-old seedlings were transferred to plastic pots containing 500 g of the peat moss/vermiculite mixture (1:1, v/v). A suspension (0.5 mL per seed) of the bacterium *Bacillus megaterium* (10^9^ cell mL^-1^) grown in Luria-Bertani medium (LB) was sprinkled over each seedling one and seven days after planting. Non-inoculated control plants received the same number of applications with the same amount of growth medium without bacteria.

### Growth conditions

Tomato plants were grown for two months in a greenhouse under controlled climatic conditions (18–24°C, with an 18 h/6 h light/dark period and 50-60% relative humidity). A photoperiod of 16 h at a photosynthetic photon flux density (PPFD) of 600 μmol m^-2^ s^-1^,as measured with a light meter (model LI-188B; Licor Inc., Lincoln, NE, USA), was maintained throughout the experiment. Water was supplied daily to maintain constant soil water content close to water –holding capacity during the entire experiment.

### Biomass production

At harvest (60 d after planting), the root system of 6 samples was separated from the shoot and fresh weight determined. Shoot and root dry weights were measured after drying in a forced draught oven at 70°C for 2 d. Plant height was also determined.

### Stomatal conductance

Stomatal conductance was recorded 2 h after dawn with the porometer system (Porometer AP4, Delta-T Devices Ltd., Cambridge, UK) in the last fully expanded leaf of six plants per treatment. Each measurement was repeated three times in each leaf, and the mean of the three measurements was considered in order to diminish variability in this parameter.

### Competition assay for colonization of tomato root tip

In order to check the ability of the examined PGPR strain to colonize wild-type and mutant plants and to confirm its presence inside roots, a competition assay for colonization was carried out. The experiment consisted of a randomized complete block design with three plant ABA line treatment: wild type, *sitiens* and *flacca* plants and two inoculation treatments: (1) control plants inoculated with LB medium alone and (2) plants inoculated with *Bacillus megaterium* strain. Six replicates of each were done totaling 36. Sterilized tomato seeds were germinated in MS plates in darkness until plants had a root 1 cm in length. 10-days-old seedlings were transferred to glass bottles containing sterile peat and vermiculite mixture (1:1, v/v). 1 mL seedling^-1^ of LB medium (control plants) or a suspension (1 mL seedling^-1^) of *Bacillus megaterium* (inoculated plants) was sprinkled over each seed at the time of transferring to bottle. *Bacillus megaterium* was grown in LB medium supplemented with gentamycin 50 μg mL^-1^ until OD_600_ = 1.9-2 that correspond to cell density of about 10^9^ cell mL^-1^. The glass bottles were kept for seven days in a controlled-climate growth chamber (18–24°C, 50-60% relative humidity, 16 h daylight) and the root systems reached an average length of 12 cm. The bacterial growth inside the roots was determined as described by Marulanda et al. [26]. A 1 cm-long distal root segment was cut, cleaned and surface-disinfected (20 min in 30% [v:v] H_2_O_2_ followed by washing in five changes of distilled water). After grinding, 100 μL aliquots were suspended in 10 mL of sterile water (dilutions 10^-2^) and 1 mL of this suspension was serially diluted to each dilution of 10^-2^ – 10^-7^. Dilutions were plated in agar nutrient broth medium (8 g L^-1^) supplemented with gentamycin 50 μg mL^-1^ and cultivated for 48 h at 28°C. Colonization experiment was repeated twice.

### Analysis of endogenous ABA

The concentration of ABA was analyzed in shoot extracts using high performance liquid chromatography-electrospray-mass spectrometry (HPLC-ESI-MS/MS). The extraction and purification of ABA were carried out using the method described by Bacaicoa et al. [27] with some variations. Frozen 0.5 g sample of plant tissue (previously ground to a powder in a mortar with liquid N) was homogenized with 5 mL of precooled (-20°C) methanol:water (80:20, v/v) and 2.5 mM Na diethyldithiocarbamate (DDTC). The deuterium-labelled internal standard [^2^H_6_] (+)-cis, trans-abscisic acid, (from Olchemim, Olomouc, Czech Republic) was added (100 μL of a stock solution of 400 ng mL^-1^ of standard in methanol) to the extraction medium. After overnight extraction at -20°C, solids were separated by centrifugation at 12000 × g for 10 min at 4°C using a Centrikon T-124 centrifuge with an A8.24 rotor (Kontron Instruments, Cumbernauld, United Kingdom) and re-extracted for 1 h with an additional 4 mL of extraction mixture. Supernatants were passed through a Strata C18-E cartridge (3 cm3, 200 mg) (Phenomenex, Torrance, CA; Ref. 8B-S001-FBJ), preconditioned with 4 mL of methanol followed by 2 mL of extraction medium. After evaporation at 40°C of aqueous phase using a Labconco Vortex Evaporator (Labconco Co., Kansas City, MO), 0.5 mL of 1 M formic acid was added. Then, ABA was extracted with two portions of 5 and 4 mL of diethyl ether, and the organic phase was evaporated to dryness. The residue was redissolved in 250 μL of methanol: 0.5% acetic acid (40:60, v/v). Before the injection in the HPLC-ESI-MS/MS system, the solution was centrifuged at 8000 × g for 5 min.

ABA was quantified by HPLC-ESI-MS/MS using a HPLC device (2795 Alliance HT; Waters Co., Milford, MA) coupled to a 3200 Q TRAP LC/MS/MS System (Applied Biosystems/MDS Sciex, Ontario, Canada), equipped with an electrospray interface. A reverse-phase column (Synergi 4 μm Hydro-RP 80A, 150 × 2 mm; Phenomenex, Torrance, CA) was used. A linear gradient of methanol (A) and 0.5% acetic acid in water (B) was used: 35% A for 1 min, 35% to 95% A in 9 min, 95% A for 4 min and 95% to 35% A in 1 min, followed by a stabilization time of 5 min. The flow rate was 0.20 mL min^-1^, the injection volume was 40 μL and column and sample temperatures was 20°C. The detection and quantification of ABA was carried out using multiple reaction monitoring (MRM) in the negative-ion mode, employing multilevel calibration curves with deuterated hormone as an internal standard. For further details see Bacaicoa et al. [27].

### Analysis of endogenous ACC content

The extraction and purification of ACC (1-aminocyclopropane-1-carboxylic acid) was carried out using the method described by Mora et al. [28]. Frozen plant tissue (0.25 g) previously ground using mortar and pestle with liquid nitrogen was homogenized with 20 μL of d_4_ACC (3 μg/mL in acetonitrile/ acetic acid 0.2% (90/10)) and 3 mL of MeOH/H_2_O/HCOOH (15/4/1, v/v/v) at -20°C. The mixture was vortexed (2000 rpm) for 10 min. After overnight extraction at -20°C, solids were separated by centrifugation (12000 rpm, 10 min, 4°C). Supernatants were purified using a Strata C18-E cartridge (Ref 8B-S001-FBJ, Phenomenex, Torrance, CA, USA) preconditioned with 4 mL of methanol and 2 mL of MeOH/H_2_O/HCOOH (15/4/1, v/v/v). The eluent was evaporated at 40°C until methanol was removed (Vortex evaporator mod. 432–2100 from Labconco Corporation, Kansas City, MO, USA). The residue was re-dissolved with 2 mL of MeOH/H_2_O/HCOOH (15/4/1) and stored at -20°C. After 1 h, the extract was newly centrifuged (12000 rpm, 10 min, 4°C). Supernatants were purified using the same Strata C18-E cartridge. After evaporation to near dryness, the residue was re-dissolved in 2 mL of formic acid 1 M, and applied to an Oasis MCK column (Ref. 186000254, Waters Co., Milford, MA) preconditioned with 4 mL of methanol and 2 mL of formic acid 1 M. The column was washed successively with 1 mL of formic acid 1 M and 1 mL of methanol. ACC was eluted with 1 mL of 0.35 M NH_4_OH. This eluted fraction was evaporated to dryness in the vortex evaporator and re-dissolved in 500 μL of acetonitrile/acetic acid 0.2% (90:10). Finally the eluted fraction was centrifuged (10000 rpm, 8 min) and injected in the LC/MS/MS systems.

ACC was quantified by HPLC linked to a 3200 QTRAP LC/MS/MS system (Applied Biosystems/ MDS Sciex, Ontario, Canada), equipped with a turbo ion spray interface. Detection and quantification were performed by multiple-reaction-monitoring (MRM) in the positive-ion mode, employing a multilevel calibration graph with deuterated d_4_ACC as internal standards. For further details see Mora et al. [28].

### Analysis of ethylene production in plant tissues

Intact plants were enclosed in sealed acetate cylinders which were incubated at room temperature for 24 h. Samples of 500 μL were withdrawn from each acetate cylinder with a syringe and the ethylene content was quantified with a Hewlett Packard model 5890 gas chromatograph equipped with a Poropak-R column and a hydrogen flame ionization detector as described Porcel et al. [29].

### RNA isolation and synthesis of first strand cDNA

Total RNA was isolated from tomato leaves from 3 different plants of each treatment by phenol/chloroform extraction method [30]. DNase treatment of total RNA and cDNA synthesis was performed according to Qiagen’s protocol (Quantitect Reverse Transcription KIT Cat#205311, Qiagen, CA).

### Quantitative real-time RT-PCR

The expression of *Solanum lycopersicum* 1-Amynocyclopropane-1-carboxylic acid oxidase (*Sl-ACO4)*, 1-Aminocyclopropane-1-caboxylic acid synthase (*Sl-ACS7)*, pathogenesis-related 1b (*Sl-PR1b)*, 9-*cis*-epoxycarotenoid dioxygenase (*Sl-NCED),* and *SlLE16* genes was studied by real-time PCR by using iCycler (Bio-Rad, Hercules, California, USA).

The primer sets used to amplify each analyzed gene in the synthesized cDNAs are shown in Table 1.

**Table 1:** Primers used in this study

		**Annealing**
**Primer**	**Sequence**	**Temperature (°C)**
SlActin For	5′ - TCACCACCACTGCTGAACGGGA-3′	58
SlActin Rev	5′ - TGGGCAACGGAACCTCTCAGC -3′	
SlPR1b For	5′ - GGGAGGGCAGCCGTGCAATT -3′	58
SlPR1b Rev	5′ - TGCAACGTGCCCGACCACAA -3′	
SlACO4 For	5′ - TTCGCGCTCACACGGATGCT -3′	58
SlACO4 Rev	5′ - CACCTCTAGCTGATCGCCGAGG -3′	
SlACS7 For	5′ - CGGTCTCCCCGGTTTTCGCA -3′	58
SlACS7 Rev	5′ - GTGGCCGCGGAGACAACCAT -3′	
SlNCED For	5′ - ACAGCCGACCCACGAGTCCA -3′	58
SlNCED Rev	5′ - GGTGTCCGGCGGTTGGTTCA -3′	
SlLE16 For	5′ - TCCCTTATCTCGAGGGTCGC -3′	58
SlLE16 Rev	5′ - CGCTGTCTTCCGGTCTTCTG -3′	

Each 23 μL reaction contained 3 μL of a dilution 1:10 of the cDNA, 10.5 μL of Master Mix (Bio-Rad Laboratories S.A, Madrid), 8.6 μL of deionised water and 0.45 μxL of each primer pair. The PCR program consisted in 3 min incubation at 95°C to activate the hot-start recombinant Taq DNA polymerase, followed by 32 cycles of 30 s at 95°C, 30 s at 58°C and 30 s at 72°C, where the fluorescence signal was measured. The specificity of the PCR amplification procedure was checked with a heat dissociation protocol (from 70–100°C) after the final cycle of the PCR.

Four independent biological replicates were used and each real-time PCR reaction was done in triplicate. These values were then normalized using the threshold cycle (C_T_) value for the tomato household gene *Sl-actin.* The relative levels of transcription were calculated by using the 2^-∆Ct^ Method [31]. Negative controls without cDNA were used in all PCR reactions.

### Statistical analysis

The data were processed by the two-way analysis of variance (ANOVA) with PGPR inoculation and plant genotype as sources of variation, using the SPSS 12.0 statistical software package, (SPSS Inc., Chicago, IL, USA). The means were considered to be significantly different at P <0.05 after the LSD test. The gene expression data were analyzed using Student’s unpaired *t* test (P < 0.05) in order to compare inoculated plants with their respective non-inoculated controls. To analyze the results of competition assay for colonization of tomato root tip, the data were subjected to the Duncan’s honestly significant difference test.

## Results

### Biomass production

**Figure 1:**
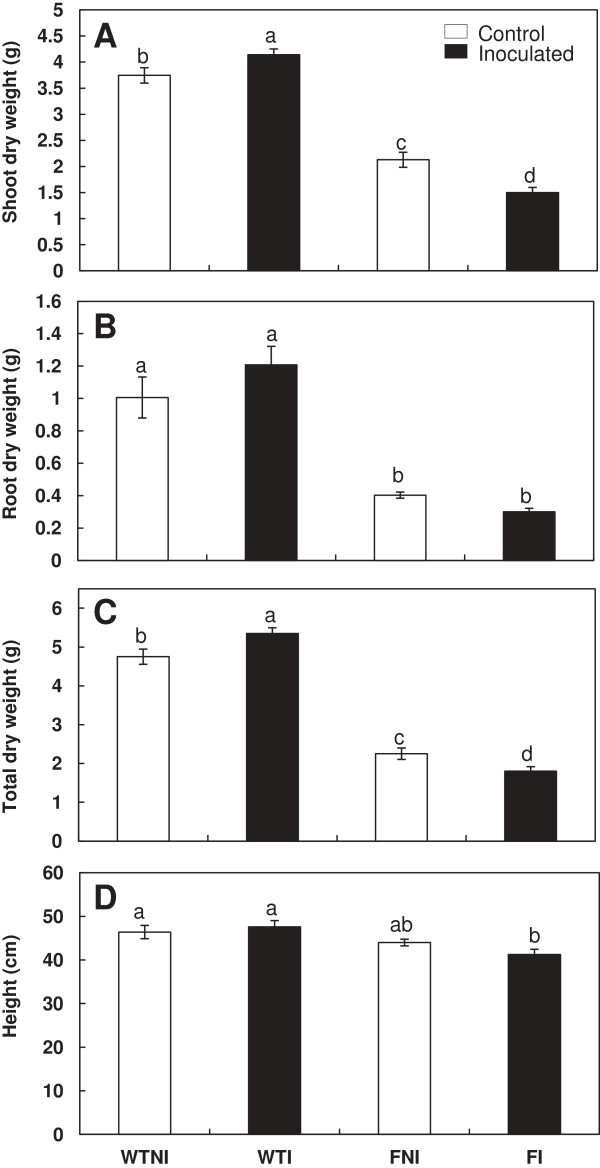
**Effects of *Bacillus megaterium *inoculation on WT and *Flacca *plant biomass.** Shoot dry weigh **(A)**, root dry weight **(B)**, total dry weight **(C)** and height **(D)** in wild-type cv Ailsa Craig (WT) and *flacca* (F) mutant tomato plants. Treatments are designed as non-inoculated controls (NI, open bars) or inoculated plants (I, black bars). Data are means ± SE (n = 6). Means followed by different letters are significantly different (P < 0.05) according to LSD’s HSD test. P values for two-way ANOVA are reported in Table 2.

**Figure 2:**
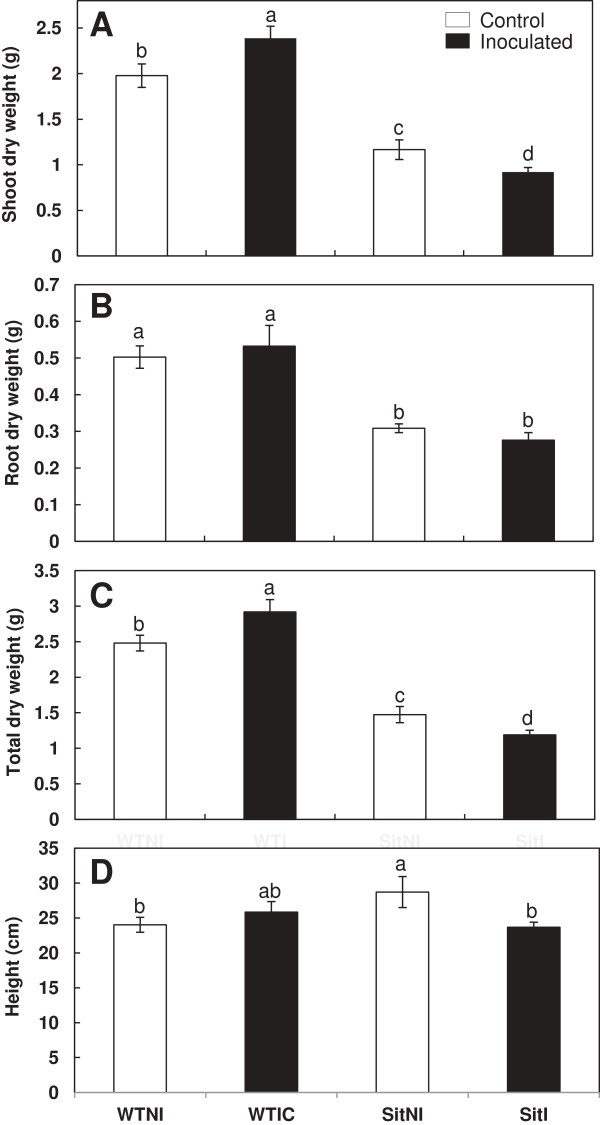
**Effects of *Bacillus megaterium *inoculation on WT and *Sitiens *plant biomass.** Shoot dry weigh **(A)**, root dry weight **(B)**, total dry weight **(C)** and height **(D)** in wild-type cv Rheinlands Ruhm (WT) and *sitiens* (Sit) mutant tomato plants. Treatments are designed as non-inoculated controls (NI, open bars) or inoculated plants (I, black bars). Data are means ± SE (n = 6). Means followed by different letters are significantly different (P < 0.05) according to LSD’s HSD test. P values for two-way ANOVA are reported in Table 2.

Shoot and root dry weight of both *flacca* and *sitiens* mutant plants followed the same pattern after inoculation with *B. megaterium* and when compared to wild-type (wt) plants. Shoot dry weight was higher in inoculated wt plants than in the corresponding non-inoculated ones (11% cv Ailsa Craig and 20% cv Rheinlands Ruhm). However, in mutant plants shoot dry weight decreased due to inoculation (30% *flacca* plants and 20% *sitiens* plants). Significant differences for shoot dry weight were found among wt and mutant plants (*flacca* and *sitiens*) regardless of microbial treatment (Figures 1A, 2A). Root dry weight was much higher in wt plants (inoculated and non-inoculated) than ABA-deficient mutant plants. No significant differences in root dry weight were observed as a consequence of inoculation with the PGPR in any plant line (both wt, *flacca* and *sitiens);* (Figures 1B, 2B).

Finally, total plant dry weight was higher in inoculated wt plants than in the corresponding non-inoculated ones (13% cv Ailsa Craig and 20% cv Rheinlands Ruhm). In contrast, in mutant plants the opposite effect was observed. Inoculation had a negative effect on total dry weight both in *flacca* and *sitiens* plants (Figures 1C, 2C).

Plant height was similar in all treatments. In the second experiment carried out with wild-type cv Ailsa Craig and *flacca* plants, only inoculated *flacca* plants showed a significant decrease in height compared with wild-type plants but not with non-inoculated *flacca* plants (Figure 1D). In the first experiment in which *sitiens* plants and their parental isogenic wild-type cv Rheinlands Ruhm were grown in other season, significant differences were observed. While in wild-type plants there was no significant change by inoculation, in *sitiens* plants a significant decrease was showed by effect of inoculation with *Bacillus megaterium* (Figure 2D). Thus, while inoculation with *B. megaterium* increased biomass in wt plants, in *sitiens* and in *flacca* plants the opposite happened.

ANOVA showed highly significant (P < 0.001) effects of genotype on shoot, root, total dry weight and height, and significant (P < 0.05) effects of genotype × PGPR interaction on shoot and total dry weight both in *flacca* and *sitiens* plants (Table 2).

**Table 2:** Two-way analysis of variance (ANOVA)

	**Significance of sources of vatiation**		
**WT/*Flacca***	**PGPR (P)**	**Genotype (G)**	**PxG**
Parameter measured			
Total DW	ns	***	**
Shoot DW	ns	***	**
Root DW	ns	***	ns
Height	ns	***	ns
Stomatal conductance	ns	***	ns
ABA	ns	***	ns
ACC	ns	***	**
Ethylene	***	ns	ns
*Sl-PR1b*	**	***	***
*Sl-ACO4*	*	ns	ns
*Sl-ACS7*	**	***	***
*Sl-NCED*	ns	***	***
**WT/*Sitiens***	PGPR (P)	Genotype (G)	PxG
Parameter measured			
Total DW	ns	***	*
Shoot DW	ns	***	*
Root DW	ns	***	ns
Height	ns	***	ns
Stomatal conductance	ns	***	ns

Significance of the sources of variation are PGPR (P), Genotype (G) and PGPR x Genotype (PxG).

*P ≤ 0.05; **P ≤ 0.01; ***P ≤ 0.001; ns, not significant.

### Stomatal conductance

**Figure 3:**
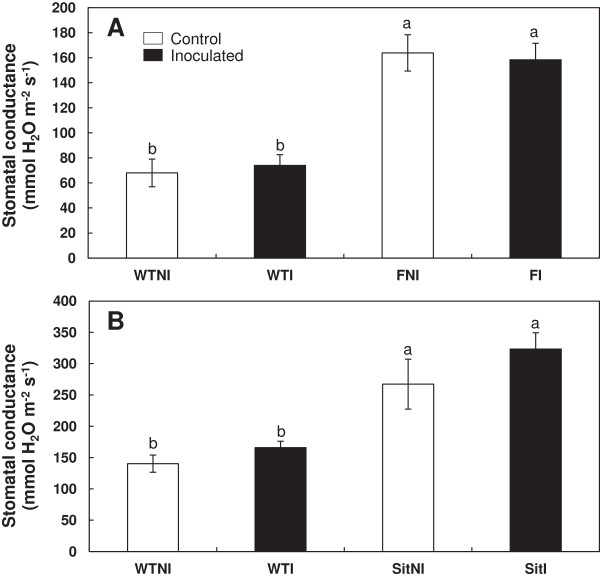
**Effects of *Bacillus megaterium *inoculation on stomatal conductance.** Wild-type cv Ailsa Craig (WT) vs. *flacca* (F) **(A)** and wild-type cv Rheinlands Ruhm (WT) vs *sitiens* (Sit) **(B)** mutant tomato plants were analyzed. Treatments are designed as non-inoculated controls (NI, open bars) or inoculated plants (I, black bars). Data are means ± SE (n = 6). Means followed by different letters are significantly different (P < 0.05) according to LSD’s HSD test. P values for two-way ANOVA are reported in Table 2.

Stomatal conductance was not affected by PGPR addition. However, *flacca* and *sitiens* mutant plants showed a much higher stomatal conductance than wt plants (Figure 3A, 3B). ANOVA analysis showed highly significant (P < 0.001) effects of genotype but not of inoculation (Table 2).

### Competition assay for colonization of tomato root tip

We carried out a competition assay for colonization in order to check the presence of *Bacillus megaterium* and its ability to colonize root wild-type and ABA-deficient mutant plants. As expected, in control non-inoculated plants we confirmed that there was no presence of *Bacillus* in roots. However, in inoculated plants, although it was present in all plants (wild-type, *sitiens* and *flacca*), the presence of *B. megaterium* was significantly lower in *sitiens* plants. Root *sitiens* plants showed 3.0, wt showed 7.4 and *flacca* plants 9.5 cfu 10^6^ cm^1^ root. Due to the similarity of results concerning biomass production and stomatal conductance, and the lower colonization of *B. megaterium* in *sitiens* plants, from now on, the following determinations were done only in *flacca* plants, where the decrease of biomass by inoculation was more pronounced.

### ABA and ACC concentration and production rate of ethylene

Since we studied whether the activity of *Bacillus megaterium* PGPR was affected by the endogenous ABA concentration of the host plant, we analyzed ABA and ACC concentration and ethylene production rate in leaves.

**Figure 4:**
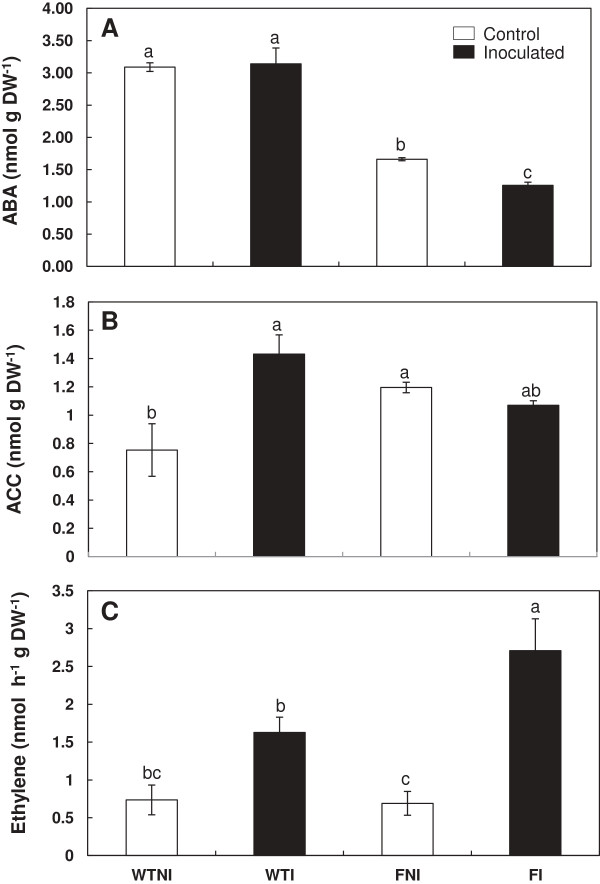
**Effects of *Bacillus megaterium *inoculation on hormones.** Concentration of shoot ABA **(A)** and ACC **(B)** and production rate of ethylene **(C)** in wild-type cv Ailsa Craig (WT) and *flacca* (F) mutant tomato plants were determined. Treatments are designed as non-inoculated controls (NI, open bars) or inoculated plants (I, black bars). Data are means ± SE (n = 6). Means followed by different letters are significantly different (P < 0.05) according to LSD’s HSD test. P values for two-way ANOVA are reported in Table 2.

Endogenous ABA concentration in leaves was lower in mutant plants than in wt plants. However, in ABA-deficient mutants, ABA decreased by 24% as a consequence of PGPR presence (Figure 4A). ANOVA showed highly significant (P < 0.001) effects of genotype (Table 2).

In leaves of wt plants, ACC concentration increased by 90% as a result of inoculation with *Bacillus megaterium*, while in leaves of mutant plants there were no significant differences as a consequence of inoculation. However, leaves of *flacca* plants showed higher intrinsic ACC concentration than wt plants (Figure 4B). ANOVA showed highly significant (P < 0.01) effects of both genotype and genotype × PGPR interaction on ACC concentration in tomato leaves (Table 2).

Ethylene production rate was increased nearly 300% in leaves of *flacca* mutants when inoculated with the PGPR. PGPR inoculation did not change significantly the ethylene production rate of wt leaves. There were no significant differences in ethylene production between non-inoculated leaves of both plant lines (Figure 4C). ANOVA also showed highly significant (P < 0.001) effects of PGPR inoculation on ethylene production rate (Table 2).

### Quantitative real-time RT-PCR

**Figure 5:**
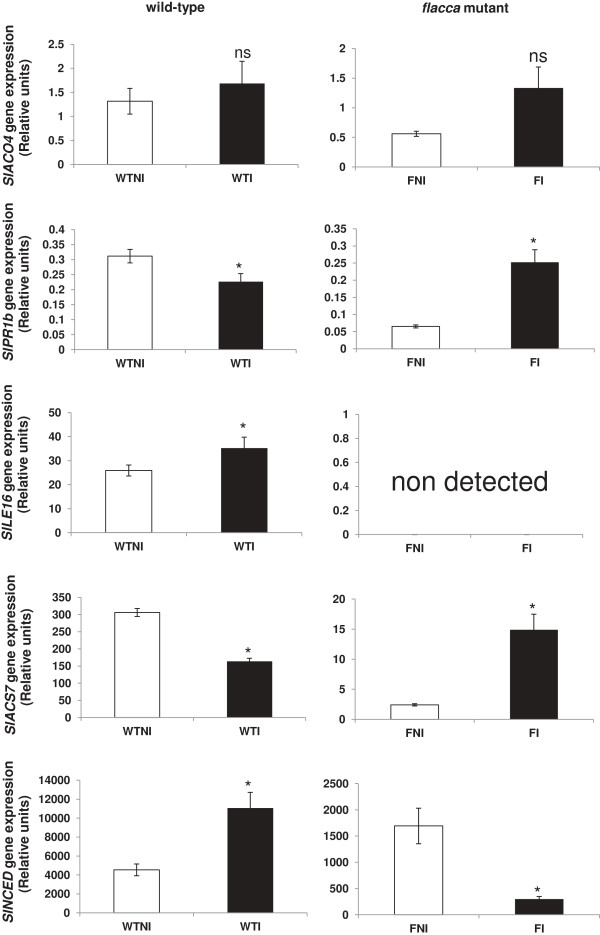
**Effects of *Bacillus megaterium *inoculation on gene expression.** Analysis of *Sl-PR1b, Sl-ACO4, Sl-ACS7, Sl-NCED* and *Sl-LE16* gene expression by real time quantitative RT-PCR in leaves of wild-type cv Ailsa Craig tomato plants and mutant *flacca* inoculated with *Bacillus megaterium*. Treatments are designed as non-inoculated plants (white bars) or inoculated wild-type or *flacca* plants (black bars). Data represent the mean values ± SE of four independent biological replicates. Means followed by asterisk are significantly different with respect to corresponding non-inoculated control (P < 0.05) as determined by t-student test. P values for two-way ANOVA are reported in Table 2.

The expression of *Sl-PR1b, Sl-ACO4, Sl-ACS7*, *Sl-NCED* and *Sl-LE16* genes was analyzed in leaves of each plant treatment (wt and *flacca* plants). *Sl-PR1b* and *Sl-ACS7* gene expression increased in leaves of ABA-deficient mutant plants in the presence of the PGPR, while in wt leaves their expression went down (Figure 5). *Sl-NCED* gene expression was shown to be inhibited in ABA-deficient mutant plants inoculated with PGPR compared with non-inoculated plants, while an important induction of gene expression was observed in inoculated wt plants compared with uninoculated wt plants (Figure 5). The data on *Sl-NCED* gene expression obtained for *flacca* mutant plants corroborated ABA-deficient phenotype in these plants since both inoculated and non-inoculated mutant plants showed a lower relative gene expression than wt ones (Figure 5). In wild-type plants *Sl-LE16* gene expression increased by inoculation but in *flacca* plants no gene expression was observed. Thus, the expression response of *Sl-PR1b*, *Sl-ACS7* and *Sl-NCED* genes to PGPR inoculation was the opposite in wt and *flacca* leaves.

ANOVA showed very significant effects (P < 0.001) of both genotype and genotype × PGPR interaction on *Sl-PR1b*, *Sl-ACS7* and *Sl-NCED* gene expression (Table 2).

## Discussion

Plant growth-promoting rhizobacteria are found in soil and when they are in association with the plant roots can stimulate the growth of the host [32]. One of the mechanisms involved in this effect is the production or degradation of hormones that regulate plant growth and development. It is known that PGPR affect root hormone concentration, and can also alter root-to-shoot long-distance signalling to mediate shoot hormonal status [33]. Several rhizobacteria produce ABA in culture media or regulate plant ABA status [17]. In fact, although the biochemical mechanisms by which ABA is produced *in planta* have been well characterized [33], there are no studies concerning the role of endogenous plant ABA on PGPR activity. Herrera-Medina et al. [34] by comparative analysis of two ABA-deficient tomato mutant plants showed that there were both quantitative and qualitative differences in the pattern of arbuscular mycorrhization colonization. ABA deficiency induced ethylene production, suggesting that one of the mechanisms used by ABA to determine susceptibility to fungal infection is through negative modulation of the ethylene pathway.

In this study, we used ABA-deficient tomato mutants (*flacca* and *sitiens*) together with their near-isogenic wt parent to study how endogenous ABA can interfere with PGPR function. *Sitiens* and *flacca* are blocked in the final step of the ABA biosynthetic pathway, where the enzyme AAO catalyses the oxidation of abscisic aldehyde to ABA [35,36]. *Sitiens* is known to have a mutation in the AAO enzyme and mutant leaves contain only c. 11% of the wild-type ABA levels [35,37]. The mutant *flacca* has a mutation in a MoCo cofactor required for the activity of AAO and mutant leaves contain c. 33% of the wild-type ABA levels [37,38]. The poor growth and strong leaf epinasty shown in the tomato mutants *notabilis*, as well as *flacca* and *sitiens* has been shown to occur even under non-wilting conditions and has been attributed, at least partially, to an excess of ethylene [39]. However, our results have shown that there are no significant differences in ethylene production rate between ABA-deficient mutant and its near-isogenic wt parental. Marulanda et al. [26] showed that the same *Bacillus megaterium* strain that was used here, had a positive effect on *Trifolium repens* growth. In the present study, this positive effect was observed in wt plants but not in *flacca* or *sitiens* ones. ABA-deficient plants showed a significant decrease in biomass production as previously demonstrated [40], indicating that endogenous ABA probably has an important role in keeping plant growth. In the same way, inoculated *flacca* and *sitiens* mutant plants showed lower heights than inoculated wt plants. Therefore, the low levels of ABA in these mutant plants switched the effect of the *B. megaterium* strain used here from plant growth promoting rhizobacteria to plant growth inhibiting rhizobacteria.

Although *B. megaterium* was present in all plants (wild-type, *sitiens* and *flacca*), the presence of this PGPR was significantly lower in *sitiens* plants. It is possible that the lower ABA may be inhibiting the root colonization. Therefore, a minimal content of ABA should be required for *B. megaterium* colonization. Similar results were found for arbuscular mycorrhizal symbiosis [34].

It has been proposed that restriction of ethylene production may be a widespread function of ABA. In fact, it is well known that ethylene and ABA act antagonistically to modulate development [41], shoot growth [39] and disease resistance in plants [42]. In addition, there is some evidence that ABA and ethylene antagonistic interaction could interfere with arbuscular mycorrhizal formation [43]. Thus, it appears that the *B. megaterium* strain used in this study may increase ethylene production of the host plant. Since *flacca* plants have lower amounts of ABA, the production of ethylene by the PGPR inoculation was exacerbated and even a reduction of the ABA levels took place.

There is a negative correlation between growth and the endogenous ABA concentration of plants [14] but not under stress conditions [44]. As we expected, ABA-deficient mutant plants showed much lower ABA concentration than wt plants, however inoculation with PGPR in these plants decreased considerably the ABA concentration in leaves, correlating with the decrease in the expression of the ABA-biosynthesis gene *Sl-NCED*. We observed a direct correlation between growth and the endogenous ABA content in *flacca* mutant plants. The hormonal response of *flacca* plants to the PGPR inoculation resembles the plant response to a pathogen, a dramatic increase in ethylene contents [45].

The *PR1b* gene is considered an indicator of plant responses to pathogens [46]. Induction of plant systemic acquired resistance (SAR) correlates with the expression of pathogenesis-related (PR) genes [47-49]. Among PR genes, PR1 expression is a paradigm for the coregulation of PR genes during SAR [50]. The interactions between ABA and ethylene in responses to plant pathogens are poorly understood. Whereas several reports have shown an inverse correlation between ABA levels and resistance to pathogens with different lifestyles in several plant species, others have suggested a positive role of this hormone in activation of defence gene expression and pathogen resistance [51]. In ABA-deficient tomato and maize ABA-biosynthesis mutants, exogenous ABA application suppresses ethylene production [41,52,53]. Consistent with this, Anderson et al. [42] found an antagonistic interaction between ethylene and ABA signalling mutants in vegetative tissues. Here, we found that in *flacca* plants with lower levels of ABA a dramatic increase in ethylene occurs with a concomitant increase in the expression of the pathogenesis-related gene *Sl-PR1b*. The results of this study are in line with the recent studies suggesting that beneficial microbes can also act as pathogens. This has been reported not only for microbes that induce systemic acquired resistance (SAR), but also for induced systemic resistance (ISR) inducers (see [54,55]). Since several studies have shown that there is a strong antagonism between ABA and salicylic acid (SA) signalling [56,57], other alternative explanation for PGPR reduction of growth in *flacca* plants could be that the ABA-deficient mutants could recognize *Bacillus* by a possible microbe-associated molecular pattern (MAMP) and could trigger a strong SA-dependent defence, leading to growth retardation.

In wt plants a significant increase in ACC concentration was observed after inoculation with PGPR, while no significant differences were observed in *flacca* mutant plants as a result of inoculation, most probably because almost all the ACC molecules were converted to ethylene in *flacca* plants. In fact, only ABA-deficient mutant plants showed a significant increase (6 times) of *Sl-ACS7,* encoding for an enzyme involved in the ethylene synthesis [58] when they were inoculated with PGPR.

## Conclusions

**Figure 6:**
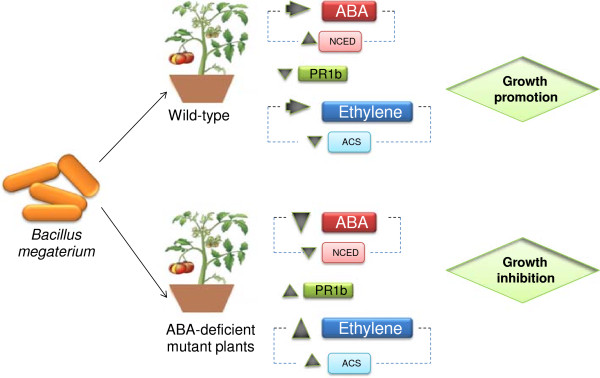
**Schematical figure showing results in wild-type and ABA-deficient mutant plants when are inoculated with *Bacillus megaterium*.** In wild-type plants, ABA concentration as well as ethylene didn’t show significant differences (compared to the non-inoculated ones). However, ABA biosynthesis-related enzyme NCED gene expression increased and ethylene biosynthesis-related enzyme ACS gene expression, decreased. In ABA-deficient mutant plants, ABA concentration as well as the enzyme involved in ABA biosynthesis gene expression, decreased. On the contrary, both ethylene and ACS gene expression increased with respect to non inoculated plants. *Sl-PR1b* gene expression was opposite in both kinds of plants. While in wild-type *Sl-PR1b* gene expression decreased, in ABA-deficient mutant plants increased significatively. In conclusion, wild-type plants inoculated with *Bacillus megaterium* showed a growth promotion while inoculated ABA-deficient mutant plants showed an inhibition of growth.

In wild-type plants, ABA and ethylene content didn’t show significant differences compared to the non-inoculated plants. However, the expression of ABA biosynthesis-related enzyme NCED gene increased and ethylene biosynthesis-related enzyme ACS gene expression decreased. In ABA-deficient mutant plants, ABA concentration as well as the enzyme involved in ABA biosynthesis gene expression decreased. On the contrary, both ethylene and ACS gene expression increased with respect to non inoculated plants. *Sl-PR1b* gene expression was opposite in both kinds of plants. While in wild-type *Sl-PR1b* gene expression decreased, in ABA-deficient mutant plants significantly increased. In conclusion, *Bacillus megaterium* stimulated growth in wild type plants, but inhibited growth in ABA-deficient mutant plants (Figure 6).

Positive correlation between over accumulation of ethylene and a higher expression of *Sl-PR1b* in ABA-deficient mutant plants could indicate that plant endogenous ABA may be essential for the growth-promoting effect of PGPR by maintaining low ethylene production levels.

## Competing interests

The authors declare that they have no competing interests.

## Authors’ contributions

RP carried out the physiological and gene expression studies. RP also performed the statistical analyses and drafted the manuscript. AMZ and JMGM carried out hormone content analyses. RA and RP conceived the study and RA moreover helped to draft the manuscript. All authors read and approved the manuscript.
